# Triplet Excited States with Multilevel Coupled Cluster Theory

**DOI:** 10.1021/acs.jctc.3c00763

**Published:** 2023-11-15

**Authors:** Sarai Dery Folkestad, Henrik Koch

**Affiliations:** † Department of Chemistry, Norwegian University of Science and Technology, Trondheim 7491, Norway; ‡ Scuola Normale Superiore, Piazza dei Cavaleri 7, Pisa 56126, Italy

## Abstract

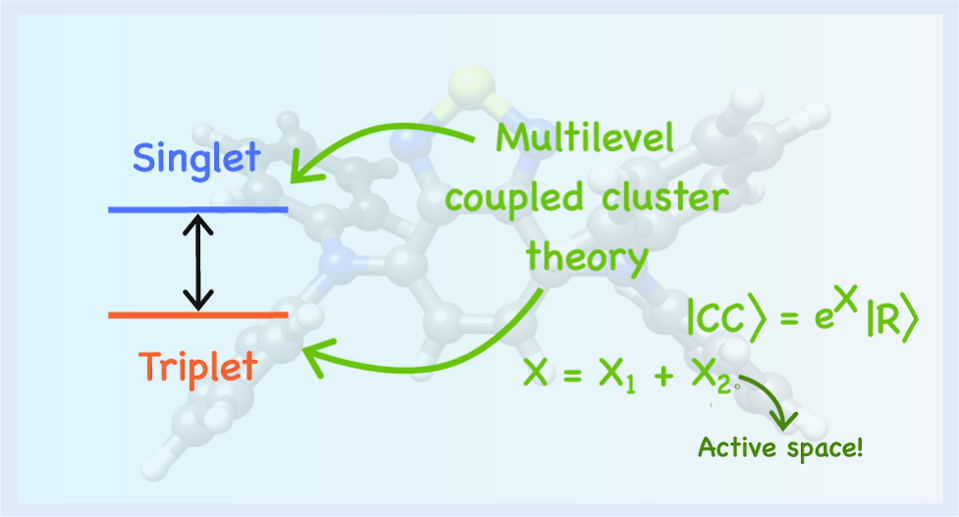

We extend the multilevel coupled cluster framework with triplet excitation energies at the singles and perturbative doubles (MLCC2) and singles and doubles (MLCCSD) levels of theory. In multilevel coupled cluster theory, we partition the orbitals and restrict the higher-order excitations in the cluster operator to a set of active orbitals. With an appropriate choice of these orbitals, the multilevel approach can give significant computational savings while maintaining the high accuracy of standard coupled cluster theory. In this work, we generated active orbitals from approximate correlated natural transition orbitals (CNTOs). The CNTOs form a compact orbital space specifically tailored to describe the triplet excited states of interest. We compare the performance of MLCCSD and MLCC2, in terms of cost and accuracy, to those of their standard coupled cluster counterparts (CC2 and CCSD) and finally show proof-of-concept calculations of the singlet–triplet gaps of molecules that are of interest for their potential use in organic light-emitting diodes.

## Introduction

The prohibitive polynomial scaling of the standard coupled cluster models has driven the development of approximate coupled cluster models with reduced cost or scaling. Multilevel coupled cluster theory is an approach to avoiding this *scaling wall*. In multilevel coupled cluster theory, higher-order excitations in the cluster operator (e.g., double or higher excitations) are restricted to an active orbital space. The approach is, simply put, obtained by setting the cluster amplitude tensors of higher order (in both ground and excited states) to zero if at least one of its indices refers to an inactive orbital. Oliphant, Adamowicz, and Piecuch^1,2^ first introduced the model to describe systems with multireference character. They were motivated by the observation that higher orders of coupled cluster theory (beyond doubles or triples) can capture both dynamic and static correlation. The approach was carried on by Piecuch and collaborators,^3^ with the CCSDt’ and CCSDt’q’ models. Köhn and Olsen^4^ recognized that the framework could generally reduce the cost of highly accurate coupled cluster calculations. Recently, Myhre and Koch^5,6^ revived the approach and coined it multilevel coupled cluster theory. They used it to model intensive properties at a significantly reduced cost.

Multilevel methods aim to describe the intensive properties of the target system. An intuitive example is the description of the core excitations of a small organic molecule in solution. Such excitation processes are localized, and localized Hartree–Fock orbitals on the solute can define the active orbital space. However, with an appropriate selection of active orbitals, the multilevel coupled cluster models can also describe delocalized intensive properties, such as charge transfer excitations.^7,8^ Multilevel coupled cluster theory has been used to calculate core and valence (singlet) excitation energies and transition moments.^6−12^ The accuracy in the intensive properties can reach that of the higher-level model, while the cost is asymptotically that of the lower-level model. For extensive properties, other methods are more suitable, such as the various local or fragmentation-based coupled cluster models, see refs (13–24) for a selection of such approaches.

The choice of active space—how many and which orbitals to use—is one of the major challenges to any active space approach. As previously mentioned, localized orbitals are suitable to describe localized intensive properties. There are many schemes to localize orbitals, such as Foster-Boys,^25^ Pipek-Mezey,^26^ or Edmiston-Ruedenberg^27^ orbitals, orthonormalized projected atomic orbitals for the virtual space,^28,29^ or Cholesky orbitals for the occupied space.^30,31^ For nonlocal excitations, correlated natural transition orbitals^32^ (CNTOs) or approximate CNTOs^7,33^ are used. The CNTOs are generated from excitation vectors and compactly describe the given transition; they are similar to the natural transition orbitals but are generated from excitation vectors with both singles and doubles amplitudes.

Triplet excited states are important in excited state dynamics, evaluation of candidates for organic light-emitting diodes, singlet fission processes, etc., and are necessary for molecular properties such as nuclear indirect spin–spin coupling. Spin-adapted triplet excited states for a variety of coupled cluster models were developed and implemented in the early 2000s.^34−36^ In this paper, we present the implementation of spin-adapted triplet excited states for CCS, CC2, CCSD, MLCC2, and MLCCSD in the  program.^37^ We also formulate CNTOs and approximate CNTOs for triplet excitations, which we use to determine the active space.

## Theory

In coupled cluster theory, the wave function is expressed through the exponential ansatz
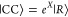
1where |*R*⟩ is a reference Slater determinant (typically the Hartree–Fock state), and *X* is the cluster operator which generates excitations of the reference
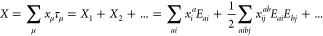
2

Here, we use the spin-adapted closed-shell formulation of coupled cluster theory, and the *E*_*ai*_ operator is a singlet excitation operator. We use *i*, *j*, *k*, ... to denote occupied orbitals, *a*, *b*, *c*, ... to denote virtual orbitals, and *p*, *q*, *r*, ... to denote general orbitals. The *x*_μ_ are the cluster amplitudes, and the τ_μ_ are excitation operators. The cluster operator is truncated at some excitation order, yielding the hierarchy of standard coupled cluster models CCS, CCSD, CCSDT, etc. Perturbation theory can be used to approximate higher excitation orders; CC2^38^ and CC3^39^ are notable examples where double and triple excitations, respectively, are included perturbatively. In multilevel coupled cluster theory, the higher excitation orders in the cluster operator are restricted to the active orbital space. In MLCC2 and MLCCSD (CCSD-in-CCS), the cluster operator is
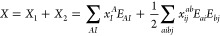
3where lower case indices are used to denote orbitals in the active space and upper case indices are both active and inactive.

The ground state coupled cluster energy and amplitudes are obtained by projection of the Schrödinger equation onto the vectors {⟨*R*|, ⟨μ|}, where ⟨μ| = ⟨*R*|τ_μ_^†^
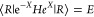
4
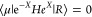
5

The nonrelativistic molecular electronic Hamiltonian
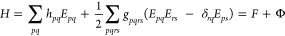
6is used. Here, *F* is the Fock operator and Φ is the fluctuation potential.^40^ Excitation energies can be obtained through linear response theory or equation-of-motion (EOM) theory. We refer the reader to ref (7). for detailed descriptions of the MLCC2 and MLCCSD ground state and singlet excited state equations.

### Triplet Excited States

Following Hald et al.,^34^ the (right) EOM-CCSD triplet excitation operator is given by
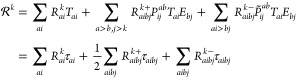
7where we have introduced the symmetrizing and antisymmetrizing operators
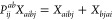
8
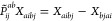
9and where τ_*aibj*_ = *T*_*ai*_*E*_*bj*_. The amplitudes *R*_*aibj*_^*k*+^ and *R*_*aibj*_^*k*–^ satisfy
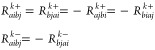
10and the operator
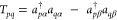
11is a triplet excitation operator.^40^ The EOM triplet excited states are then defined as
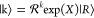
12and can be determined by diagonalizing the similarity transformed Hamiltonian in the biorthonormal triplet basis^34^
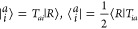
13
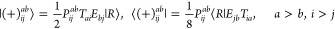
14
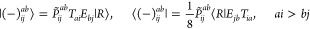
15

The (right) eigenvectors of the similarity transformed Hamiltonian in this basis are the states in eq 12, and the eigenvalues are the triplet energies.

Subtracting the ground state energy from the diagonal of the similarity transformed Hamiltonian in the triplet basis yields the triplet Jacobian matrix, whose eigenvalues are the excitation energies (ω^*k*^)
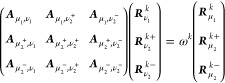
16

The triplet CCSD Jacobian matrix has the form
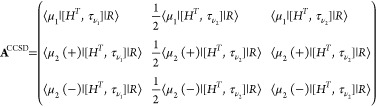
17and the explicit expressions for the linear transformation by this matrix can be found in ref (34).

If we solve the doubles equations from eq 16
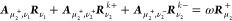
18
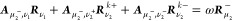
19to first order in the fluctuation potential Φ—defining the single amplitudes (ground- and excited state) as zeroth order in the perturbation and the doubles amplitudes (ground- and excited state) as first order in the perturbation—we obtain the EOM-CC2 triplet equations. The triplet CC2 Jacobian matrix has the following form
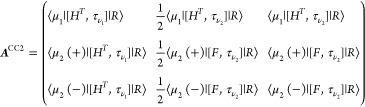
20

Because of the symmetries of  and , we have in CC2
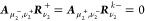
21

Note that the EOM excitation energies are the same as those obtained with the linear response theory. In MLCC2 and MLCCSD for triplet excited states, we restrict the doubles amplitudes and basis vectors to the active space, and the Jacobian matrices are straightforwardly obtained from the CC2 and CCSD triplet Jacobian matrices.

In the following section, we will demonstrate that (for a fixed active space) the MLCC2 and MLCCSD equations scale as  with the system size. The -scaling terms appear at the CCS level of theory.

### Linear Transformation by the Triplet Jacobian Matrix

The linear transformation by the triplet Jacobian matrix is given by
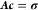
22

For models with explicit double excitation parameters in the excitation vectors, both ***c*** and **σ** have the shape
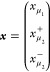
23where ***x***^+^ and ***x***^–^ display the symmetries given in eq 10. We will sometimes use the vector
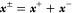
24

We use a *X*_1_-transformed Hamiltonian^40^
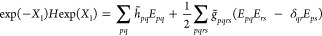
25where *g~* and *h~* are *X*_1_-transformed one- and two-electron integrals defined as
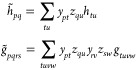
26and
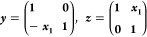
27and ***x***_**1**_ is the rectangular matrix of the single excitation cluster amplitudes.^40^

The *X*_1_-transformed electron repulsion integrals are factorized using a Cholesky decomposition
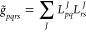
28

For the *X*_1_-transformed integrals, we have
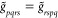
29
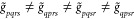
30and therefore the Cholesky vectors, ***L***^*J*^, are not symmetric in the *X*_1_-transformed basis. We will also use the intermediates
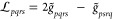
31
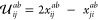
32and
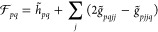
33Equation 33 defines the Fock matrix in the *X*_1_-transformed basis.

We will now consider the scaling of the MLCC2 and MLCCSD equations with the system size for a small fixed active space.

#### MLCC2

The linear transformation by the MLCC2 triplet Jacobian is obtained by inserting eq 20 into eq 22 and restricting all doubles amplitudes to the active space. We obtain
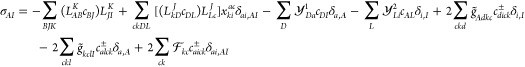
34where we have introduced the intermediates
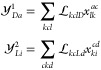
35

The first term of eq 34 enters at the CCS level of theory and scales as  with the system size. If we consider a fixed active space, the second term scales as  and the next four terms scale as . The last term does not scale with the system size when the active space is fixed. In the -program,^37^ the two-electron integrals are generally constructed on the fly from the Cholesky vectors. In a standard Cholesky factorization, the number of Cholesky vectors is proportional to the number of AOs.^41^ Hence, the construction of a block of  with one unrestricted index scales as . Construction of the intermediates in eq 35 therefore scale as  with a fixed active space but need only be calculated once.

Although only the active part of the occupied-virtual block of  enters the MLCC2 equations, the full occupied-virtual block is constructed; the construction scales as  but is done only once. The operation can be reduced to  scaling if only the active block is calculated.

For the double-excitation part of the linear transformation, we have
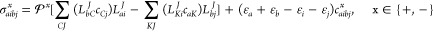
36where ε_*p*_ is a diagonal element of the active Fock matrix and
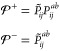
37with *P*_*ij*_^*ab*^ and  defined in eqs 8 and 9, and
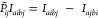
38

The first two terms scale as  when the active space is fixed, and the last term does not scale with the system size.

#### MLCCSD

The single excitation part of **σ** is the same in MLCC2 and MLCCSD. For the double-excitation parts, we have the contributions (for *x* ∈ {+, −})
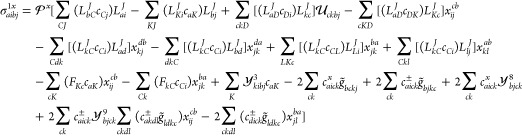
39
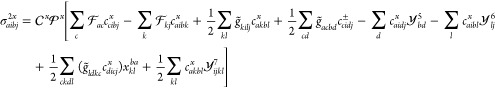
40and
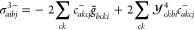
41

The MLCCSD linear transforms are given by
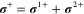
42
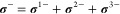
43

Here we have introduced the coefficients  and  and the intermediates
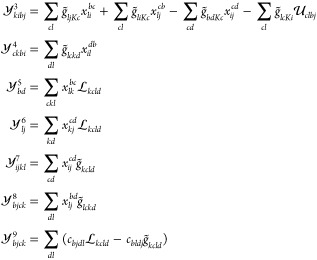
44which are calculated only once. In eq 39, there are a few terms which scale as  if the active space is fixed; these become the most expensive terms when the inactive space becomes significantly large. The most expensive contraction in CCSD (the term which scales ) does not scale with the system size in MLCCSD, except that the construction of the integral *g*_*acbd*_ scales as  due to the use of Cholesky vectors. As was the case for MLCC2, the terms that scale most severely with the full system size are the terms arising at the CCS level of theory and the construction of .

### Correlated Natural Transition Orbitals for Triplet States

At the CCSD level of theory, the triplet excitation vector has the form . The excitation vector is normalized such that
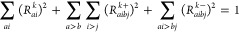
45which can be written in terms of unrestricted summations
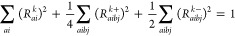
46

Following the definition of the CNTOs for singlet excited states, we define CNTO transformation matrices according to
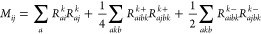
47
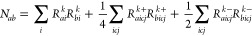
48ensuring that Tr(*M*) = Tr(*N*) = 1. The trace conditions on the matrices mean that the eigenvalues of ***M*** and ***N*** sum to 1. As suggested by Høyvik et al.,^32^ the number of CNTOs that generate the active orbital space for the MLCC calculations can be determined from the condition
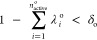
49
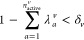
50

The eigenvalues of ***M*** and ***N*** (λ_*i*_^o^ and λ_*a*_^v^) are added in descending order.

If an MLCC calculation is performed for several states simultaneously, the CNTOs can be generated by diagonalizing the sum of ***M***- and ***N***-matrices obtained from a lower-level calculation of each of the excited states (*k*)
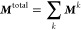
51
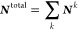
52

The trace condition for the calculation of *n* states will then become Tr(***M***^total^) = Tr(***N***^total^) = *n*. To determine the size of the active space, we must take this into account and scale the eigenvalues of ***M***^total^ and ***N***^total^ by 1/*n*.^7^

In MLCC2 and MLCCSD (CCSD-in-CCS) calculations, only CCS triplet excited states are available to generate active orbital space. Since the NTO ***N***-matrix has a rank lower or equal to *n*_o_, NTOs cannot provide a suitable virtual active space for MLCC2 or MLCCSD calculations. Therefore, we introduce approximate double excitation vectors according to
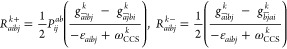
53where ε_*aibj*_ is the orbital energy difference, ω_CCS_ is the CCS excitation energy, and where we have defined
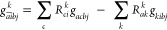
54in terms of the triplet single excitation vector with elements *R*_*ai*_. These expressions are obtained through the perturbative treatment of double excitations in the description of triplet excited states, see ref (42). Approximate CNTOs were introduced by Baudin and Kristensen for singlet excited states.^33^

Using these expressions for ***R***_2_^*k*+^ and ***R***_2_^*k*–^, and the ***R***_1_^*k*^ vector from a CCS calculation, we may construct approximate CNTOs using eqs 47 and 48. However, proper normalization according to eqs 45 and 46 must first be enforced.

The calculation of eq 54 has an  cost, and the construction of approximate CNTOs will become the computational bottleneck of an MLCC2 or MLCCSD calculation. In ref (8), we demonstrated this for singlet excitations, and the picture remains the same for triplet excited states. There are several ways to avoid this cost. One alternative is to use localized orbitals rather than CNTOs. Localized orbitals can be obtained through  operations.^25,26^ However, the quality and compactness of such an active space are generally reduced compared to those obtained from CNTOs. Alternatively, for large molecular systems where a single  is impractical, a two-step procedure can be used where active localized orbitals are used to generate reduced space CCS excitation vectors, which in turn are used to construct approximate CNTOs. A final solution exploits that the NTOs are primarily inadequate to determine the virtual space: First, an ***M***-matrix generated from CCS excitation vectors can be used to make an initial selection of the active occupied space. The resulting reduced occupied space can be used to generate approximate doubles, a procedure which is  if the occupied space does not scale with the system size. The quality of the resulting CNTOs will be benchmarked in a future publication.

Although the CNTOs are used to determine the active space for the MLCC calculations, the orbitals used in the actual MLCC calculation are obtained by block diagonalizing the occupied–occupied and virtual–virtual Fock matrices in the active–active and inactive–inactive blocks, as detailed in ref (7). This transformation leaves the active (and inactive) orbital spaces unchanged but yields an orbital basis where the diagonal elements of the Fock matrix can be used to replace the orbital energies that are central to standard canonical coupled cluster algorithms. For MLCC2 and MLCCSD, the purpose of this transformation is 2-fold: first, the standard quasi-Newton approach for solving the coupled cluster equations uses the orbital energy differences as the approximate Jacobian. Second, for canonical CC2, an analytical expression for the doubles amplitudes exists. By block diagonalizing the Fock matrix and using the diagonal elements in place of the orbital energies, the convergence of the ground-state MLCC equations and a closed-form expression for the doubles amplitudes in MLCC2 are retained.

## Results

All calculations have been carried out with an optimized implementation in a development version of the  program.^37^ The Hartree–Fock equations are converged to a maximum norm of 10^–7^, the Cholesky decomposition threshold is 10^–4^, and the ground and excited state coupled cluster equations are converged to a residual norm (*l*_2_-norm) of 10^–5^ and 10^–3^, respectively. These are all default thresholds of  v1.9. Molecules are visualized using the Chimera software package.^44^

### Cost and Accuracy

**Figure 1:**
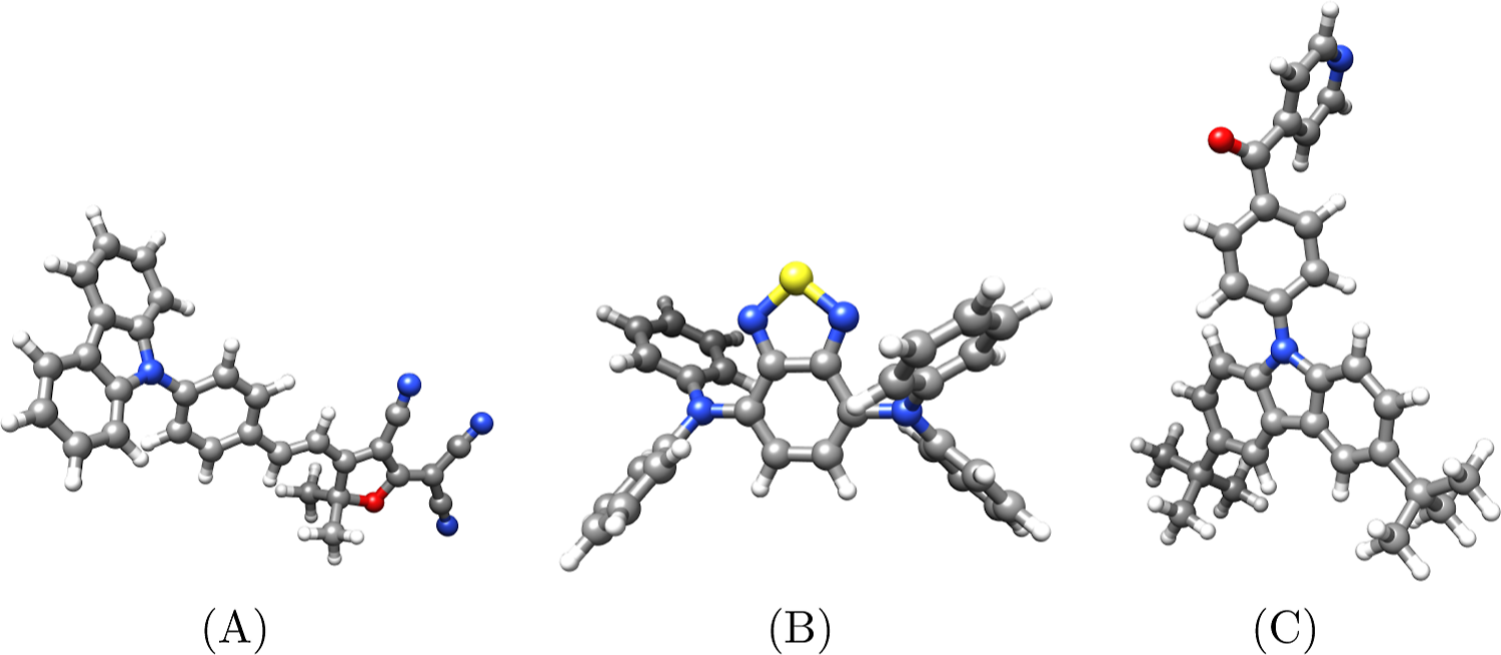
Molecules A–C considered in this study, geometries are obtained from the Supporting Information of ref (43)

To demonstrate the performance of MLCC2 and MLCCSD for triplet excitation energies, we consider the molecule shown in Figure 1a. The geometry is taken from the Supporting Information of ref (43).

**Figure 2:**
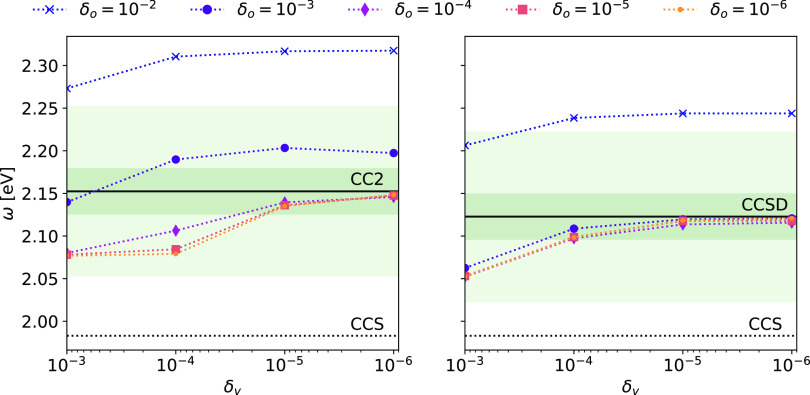
Convergence of the lowest MLCC2 (left) and MLCCSD (right) triplet excitation energies of molecule A for different CNTO thresholds. Each line corresponds to an occupied threshold δ_o_, and the virtual threshold is on the *x*-axis. Shaded areas indicate the 0.1 eV and 1 mEh deviations from the CC2 and CCSD energies.

In Figure 2, we show the convergence of the lowest MLCC2 and MLCCSD excitation energies to the CC2 and CCSD excitation energies with increasing active space size. For MLCCSD, we obtain mE_h_ errors compared to those of CCSD with δ_o_ ≤ 10^–3^ and δ_v_ ≤ 10^–4^. For MLCC2, tighter thresholds were needed, and m*E*h errors compared to CC2 are obtained with δ_o_ ≤ 10^–4^ and δ_v_ ≤ 10^–5^. However, both with MLCC2 and MLCCSD, an error of approximately 0.1 eV (∼3.8 mE_h_) compared to the appropriate standard models is acceptable, since the errors of CCSD and CC2 are of this order. For this system, errors below 0.1 eV are obtained with MLCC2 and MLCCSD when δ_o_, δ_v_ ≤ 10^–3^.

**Figure 3:**
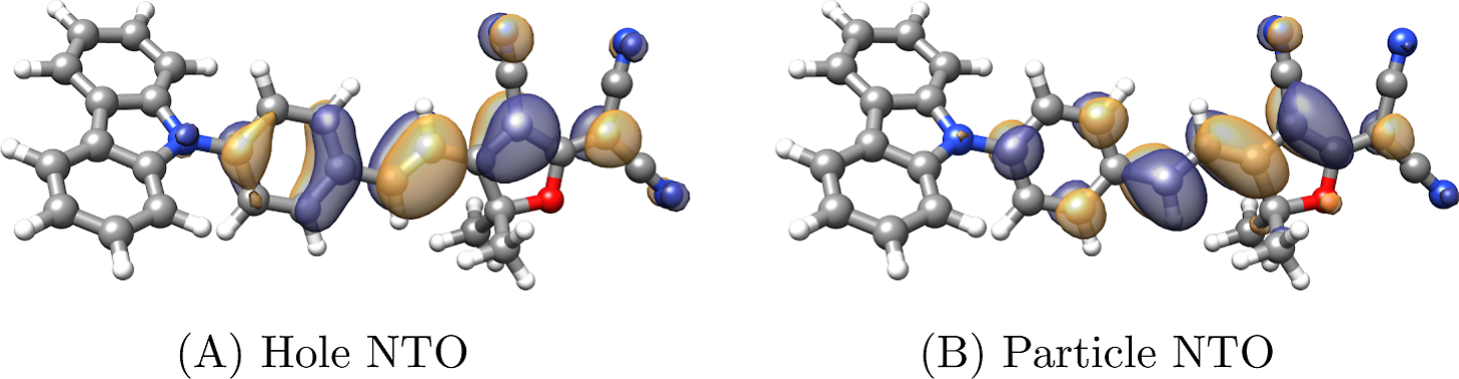
NTOs for the lowest triplet CCS excitation of molecule A. The corresponding eigenvalue is 0.84 and the isovalue is 0.03 a.u.

In Table 1, we present the lowest triplet excitation energy (ω_*T*_) obtained with MLCC2 and MLCCSD for different CNTO thresholds δ_o_ = δ_v_. We compare to the CCS, CC2, and CCSD excitation energies. We also list the average wall (*t̅*_W_) and CPU (*t̅*_CPU_) times required to transform vectors by the triplet Jacobian matrices of the different models. The MLCC2 wall times are compared to the CC2 wall times, and MLCCSD is compared to CCSD. Significant savings can be obtained with the multilevel approach compared to the standard models. In particular, we note that, for this system, an error smaller than 1 mE_h_ is obtained in a calculation that is more than 70 times faster than the full CCSD calculation (δ_o_ = δ_v_ = 10^–4^). We note that this excitation is delocalized; this is evident from the NTOs of the CCS triplet excitation shown in Figure 3, demonstrating that the approach is not limited to localized excitation processes because CNTOs are used to generate the active space.

**Table 1:** Calculations of Molecule A Using the aug-cc-pVDZ Basis^a^

model	δ_o_/δ_v_	*n*_o_^a^	*n*_v_^a^	ω_*T*_ [eV]	*t̅*_CPU_ [s]	*t̅*_W_ [s]	*t̅*_W_/*t̅*_W_^ref^
CCS		83	866	1.983	23.22	0.33	
MLCC2	10^–3^/10^–3^	38	103	2.140	156.51	2.05	9 × 10^–3^
	10^–4^/10^–4^	61	270	2.106	993.20	14.10	6 × 10^–2^
	10^–5^/10^–5^	76	460	2.136	3389.52	57.46	0.26
	10^–6^/10^–6^	82	632	2.148	6431.90	106.70	0.48
CC2		83	866	2.152	12721.55	224.49	
MLCCSD	10^–3^/10^–3^	38	103	2.068	297.50	3.96	7 × 10^–4^
	10^–4^/10^–4^	61	270	2.098	5807.20	80.14	1.3 × 10^–2^
	10^–5^/10^–5^	76	460	2.118	42777.30	611.71	0.11
	10^–6^/10^–6^	82	632	2.121	124558.80	1685.01	0.29
CCSD		83	866	2.123	388772.70	5740.75	

a The lowest triplet excitation is given, together with the average wall (*t̅*_W_) and CPU time (*t̅*_CPU_), to transform a single vector by the triplet Jacobian matrix. For the MLCC models, we used δ_o_ = δ_v_ for the CNTO thresholds. The calculations are performed on Intel Xeon Platinum 8380 CPUs, using 80 threads. 500 GB of memory was used, except in the CCSD calculation, where 750 GB was used.

**Figure 4:**
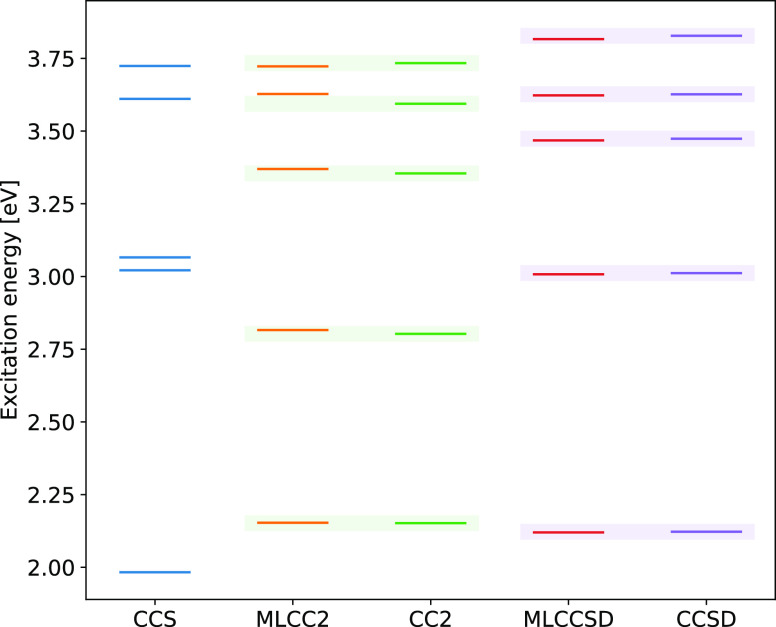
Five lowest triplet excitation energies of molecule A, as obtained with CCS, CC2, CCSD, MLCC2, and MLCCSD. The MLCC calculations are performed with CNTO thresholds of δ_o_ = δ_v_ = 10^–4^. The 1 mEh deviations with respect to CC2 and CCSD are indicated by shaded areas.

To calculate several triplet states, there are two options for MLCC with CNTOs. One can calculate each state separately, generating a different active space for each of the excited states. This procedure gives excitation vectors that are not orthogonal, and transition properties between the excited states cannot be calculated in a straightforward manner. Alternatively, one can calculate a common active space for all of the states. This procedure will give less computational savings since the active space necessarily will be less compact but will make the calculation of transition properties easier. In Table 2, we present the timings for the triplet Jacobian transformations when the five lowest excited states are used to generate common CNTOs. Thresholds of δ_o_ = δ_v_ = 10^–4^ are used. The occupied and virtual active spaces increase compared to the calculations of a single excited state (Table 1), and consequently, the cost also increases. However, significant computational savings are still obtained. In Figure 4, we can see that the MLCC energies are generally within 1 m*E*_h_ of the standard models in these calculations but that the errors are larger for MLCC2.

**Table 2:** Timings to Obtain the Five Lowest Triplet Excited States of Molecule A Using the aug-cc-pVDZ Basis^a^

model	*n*_o_^a^	*n*_v_^a^	*t*_CPU_ [s]	*t*_CPU_ [s]	*t*_W_/*t*_W_^ref^
CCS	83	866	21.18	0.28	
MLCC2	77	368	2428.66	37.006	0.19
CC2	83	866	11950.21	198.78	
MLCCSD	77	368	25514.84	344.88	0.07
CCSD	83	866	365328.66	5033.47	

a Average wall (*t̅*_W_) and CPU time (*t̅*_CPU_) to transform a single vector by the triplet Jacobian matrix are given. The CNTO thresholds are δ_o_ = δ_v_ = 10^–4^. The calculations are performed on Intel Xeon Platinum 8380 CPUs, using 80 threads, and 1 TB of memory was available.

### Scaling

**Figure 5:**
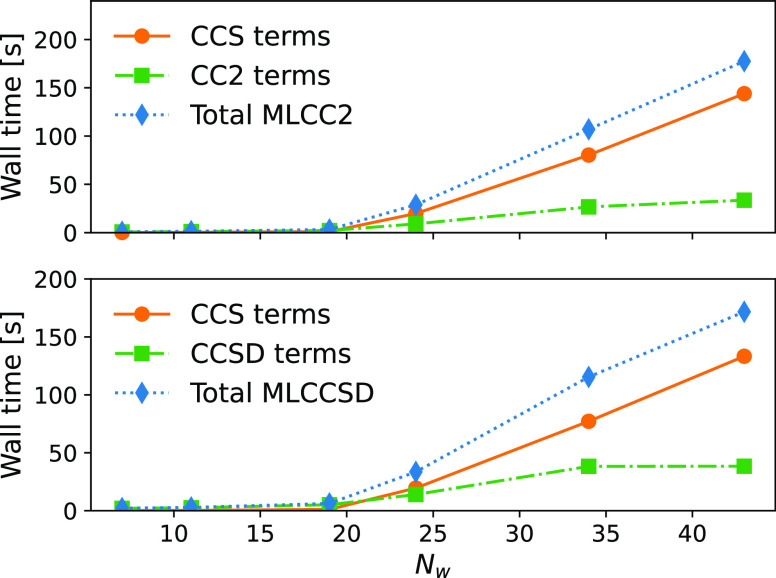
Paranitroaniline and water: wall time contributions to the MLCC2 and MLCCSD triplet Jacobian transformations from terms associated with the CCS and CC2 or CCSD levels of theory.

**Figure 6:**
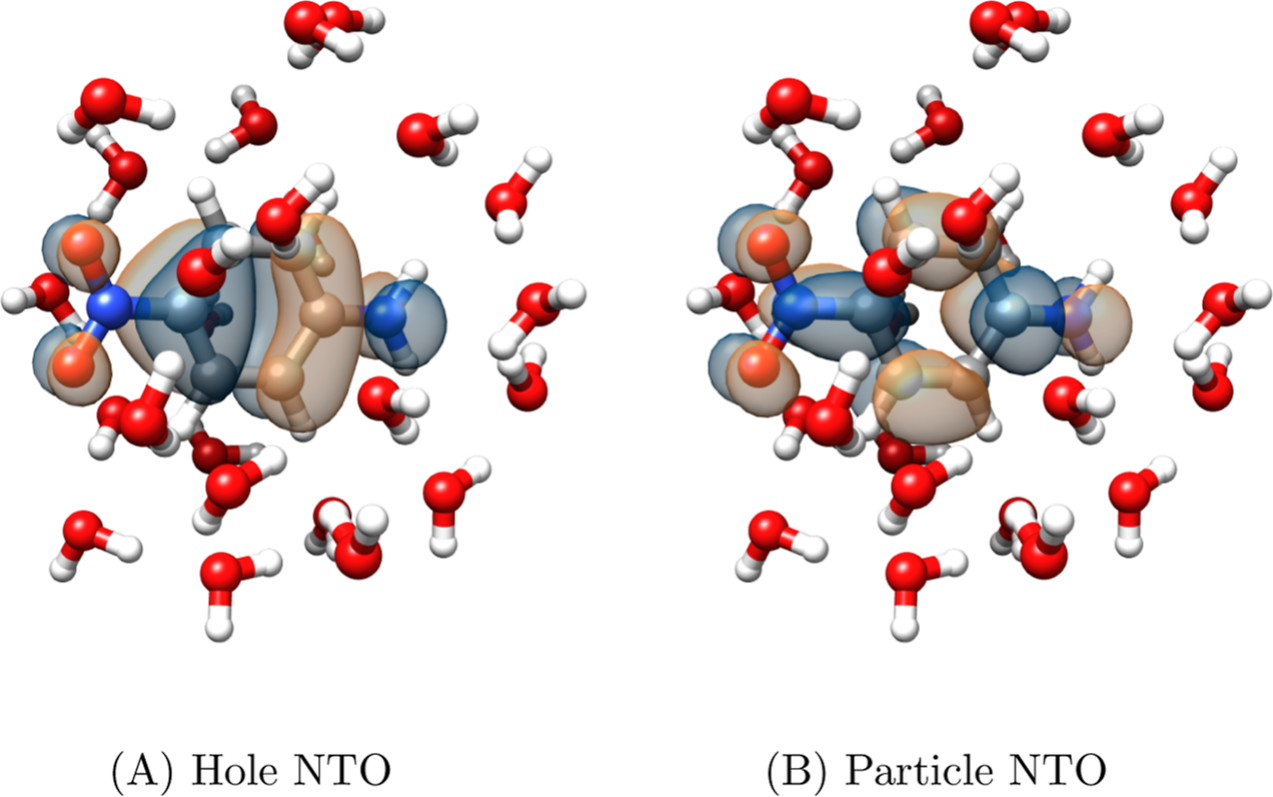
MLCC2 NTOs for the lowest triplet excitation of PNA and 24 water molecules. For all PNA-water geometries, the MLCC2 NTOs have the same character. Isovalue 0.03 au.

With a sufficiently small active space (relative to the full system size), the scaling of the multilevel coupled cluster calculation will be that of the lower-level model. In Figure 5, we consider the wall times for the Jacobian transformations to obtain the lowest triplet excitation of paranitroaniline (PNA) in water (Figure 6b). The geometries are given in ref (45). The active space contains around 12 occupied orbitals and 120 virtual orbitals. Which corresponds to CNTO thresholds of δ_o_ = 10^–3^, δ_v_ = 10^–4^ for the smaller systems. The contribution to the wall time from terms arising at the CCS level of theory becomes dominating as the inactive space becomes larger (more water molecules included in the calculation); the -scaling of the CCS-terms in eq 34 determines the asymptotic scaling of an MLCC2 or MLCCSD triplet calculation. From Figure 6, we can see that the excitation in question is localized on the PNA molecule.

### Illustrative Calculations of Singlet–Triplet Gaps

Finally, the singlet–triplet gaps of the molecules in Figure 1 are given in Table 3. These molecules have been studied for their prospects in organic light-emitting diodes.^43^ We compare the CCS, CC2, CCSD, MLCC2, and MLCCSD excitation energies. For all calculations, CNTO thresholds of δ_o_ = 10^–3^ and δ_v_ = 10^–4^ are used in both singlet and triplet calculations. Singlet CNTOs and their use in MLCC2 and MLCCSD have been described in refs (32) and (7). All MLCC2 and MLCCSD calculations have errors well within the 0.1 eV limit (compared to those of CC2 and CCSD). However, since the errors in the singlet and triplet excitations are sometimes of opposite sign, the error in the singlet–triplet gap can become larger, and tighter CNTO thresholds might, generally, be required to obtain higher accuracy in this property. The CC2 results agree with the results in ref (43), where they calculated RI-CC2 triplet excitation energies of these molecules, including the effect of a cyclohexane solvent with the polarizable continuum model.

**Table 3:** Lowest Singlet and Triplet Excitation Energies (ω_S_ and ω_T_), and the Singlet Triplet^a^ Gap (Δ_S–T_ = ω_S_ – ω_T_) of the Molecules in Figure 1^a^

	molecule A			molecule B			molecule C		
model	ω_S_	ω_T_	Δ_S–T_	ω_S_	ω_T_	Δ_S–T_	ω_S_	ω_T_	Δ_S–T_
CCS	3.83	1.98	1.85	3.55	1.84	1.71	4.83	3.04	1.79
MLCC2	2.65	2.19	0.46	2.42	2.12	0.30	3.52^b^	3.23	0.29
CC2	2.69	2.15	0.54	2.44	2.05	0.39	3.48^b^	3.20	0.28
MLCCSD	3.23	2.11	1.12	2.86	2.07	0.79	3.90	3.22	0.68
CCSD	3.29	2.12	1.17	2.91	2.09	0.82	3.89	3.30	0.58

a All MLCC calculations are performed with CNTO thresholds of δ_o_ = 10^–3^ and δ_v_ = 10^–4^. All energies are given in eV.

b Lowest excitation has different character than that of CCS and CCSD. The CC2 (and MLCC2) state, corresponding to the state found in CCS and CCSD, has 3.71 eV at both CC2 and MLCC2 levels of theory.

## Concluding Remarks

We report the implementation of triplet excitation energies within the multilevel coupled cluster framework. The study of triplet excited states is, for instance, important for materials that exhibit intersystem crossings, phosphorescence, or thermally activated delayed fluorescence; the latter being a central process in organic light-emitting diodes. Often, molecular systems relevant for use in organic light-emitting diodes are too large to be studied with standard coupled cluster models. We show that the MLCC2 and MLCCSD models can provide significant computational savings while high accuracy (<0.1 eV error to standard CC2 or CCSD) is retained. The asymptotic scaling for a fixed active space is ; the same scaling as a CCS calculation. To illustrate the benefits of a multilevel coupled cluster approach to triplet excitation energies, we have calculated the singlet–triplet gaps of molecules that are of interest for their potential use in organic light-emitting diodes.^43^ These molecules are large enough that the routine application of CCSD is impractical. With MLCCSD, they can be considered with ease.

The quality of multilevel coupled cluster calculations relies on the selection of active orbitals. Correlated natural transition orbitals (CNTOs) contain information about an excitation process.^32^ They have been used, with success, for singlet excited states within a multilevel coupled cluster framework. Here, we formulate CNTOs for triplet excited states as a straightforward extension of singlet CNTOs. Using excitation vectors from a CCS calculation and approximating the contribution from double excitations, a compact active space can be generated for MLCC2 and MLCCSD calculations.

The developments presented here extend the application range of the multilevel coupled cluster models and are a step toward a description of essential properties that depend on the triplet excited states of the molecule.
